# Effects of blue light during and after exposure on auditory working memory

**DOI:** 10.1186/s40101-025-00395-6

**Published:** 2025-05-22

**Authors:** Kyungshil Kim, Koichi Yokosawa, Ken Okada, Hayate Onishi, Yumiko Tan, Sang-il Lee

**Affiliations:** 1 Faculty of Medical and Health Sciences, Hokkaido Bunkyo University, 196-1 Kogane-Chuo 5-Chome, Eniwa, Hokkaido 061-1449 Japan; 2 Faculty of Health Sciences, Hokkaido University, Kita 12, Nishi 5, Kita-Ku, Sapporo, Hokkaido 060-0812 Japan; 3 Graduate School of Engineering, Hokkaido University, Kita 13, Nishi 8, Kita-Ku, Sapporo, Hokkaido 060-0813 Japan; 4 Graduate School of Health Sciences, Hokkaido University, Kita 12, Nishi 5, Kita-Ku, Sapporo, Hokkaido 060-0812 Japan; 5 Faculty of Engineering, Hokkaido University, Kita 13, Nishi 8, Kita-Ku, Sapporo, Hokkaido 060-0813 Japan

**Keywords:** Blue light, Auditory working memory, Phonological loop, Cognitive function, Human

## Abstract

**Introduction:**

Exposure to short-wavelength light (i.e., blue light) has been shown to enhance cognitive function in humans. While most prior studies have focused on visuospatial working memory, the effects of blue light on auditory working memory, particularly tasks involving the phonological loop, remain underexplored. This study investigated both the during- and post-exposure effects of blue light on auditory memory performance.

**Methods:**

Fifteen healthy university students (13 males, 2 females; 21.47 ± 1.06 years old) participated in a randomized crossover design. Each participant was exposed to three lighting conditions for approximately 20-min: blue (λ_max_ = 476 nm, illuminance = 21.84 lx, 13.8 log photons/s-1.cm-2, melanopic EDI = 169.68 lx), amber (λ_max_ = 580 nm, illuminance = 61.65 lx, 13.5 log photons/s-1.cm-2, melanopic EDI = 2.87 lx) and dim light (baseline; illuminance < 5.00 lx). Each session was separated by a one-week washout period. To mitigate order effects, the sequence of light conditions was randomized across participants. The modified version of the Sternberg working memory task was performed during light exposure and after a 10-min break (i.e., During- vs Post-exposure phase). The accuracy, reaction time, subject anxiety and subject sleepiness were measured.

**Results:**

In the post-exposure phase, blue light significantly improved accuracy compared to amber (*p* < 0.01, d = 0.66) and dim light (*p* < 0.01, d = 0.67). No significant differences were observed during exposure or in reaction time across three light conditions. Anxiety levels were significantly higher during blue light exposure (vs. amber: *p* = 0.013, d = 0.96; vs. dim: *p* = 0.027, d = 0.83), while sleepiness remained unchanged.

**Conclusions:**

Blue light exposure may enhance auditory working memory accuracy with a delayed effect, independent of vigilance or processing speed. While these findings are promising, the observed effects are preliminary and require validation in larger and more diverse populations.

## Introduction

Working memory, as described by Baddeley (2012) [1], is a complex cognitive system that is believed to be necessary for temporarily holding information while performing complex tasks such as reasoning, comprehension, and learning. This system comprises multiple components, including visuospatial sketchpad, phonological loop, episodic buffer, and central executive system [1]. The visuospatial sketchpad stores and manipulates visual and spatial information, aiding in mental imagery [1, 2]. This subsystem is primarily mediated by neural circuits encompassing the right dorsolateral prefrontal cortex, posterior parietal cortex, and occipital regions, which are involved in visual processing and spatial attention [3, 4] The phonological loop retains and rehearses phonological information and plays a key role in language acquisition and communication skills [1, 5]. It involves brain regions such as Broca’s area (typically situated in the left inferior frontal gyrus), Wernicke’s area (typically situated in the left superior temporal gyrus), and the supramarginal gyrus, all of which are engaged in speech perception, articulation, and phonological processing [3, 4]. Irregularities within the phonological loop are associated with symptoms related to language and learning disorders [1, 6]. The episodic buffer integrates information from various sources in a limited-capacity storage [1]. The central executive system integrates information from the visuospatial sketchpad and the phonological loop and accesses long-term memory for problem-solving and decision-making [1].

Exposure to short-wavelength light (i.e., blue light) has been shown to improve cognitive function and regulate mood in humans [7–16]. The activity of intrinsically photosensitive retinal ganglion cells (ipRGCs), which are highly sensitive to short-wavelength light [17], might be responsible for the beneficial effects of blue light exposure on cognitive performance. These cells are known to regulate non-image-forming responses such as circadian photoentrainment, pupillary light reflex, and the regulation of melatonin secretion [18]. Furthermore, recent evidence indicates that ipRGCs contribute to cognitive processes such as attention and working memory, potentially through direct projections to the prefrontal cortex (PFC) [16, 19]. Previous research has predominantly focused on the effects of blue light on visuospatial working memory. For instance, Alkozei et al. (2016) [10] found that blue light exposure enhances response time and PFC activity during a visuospatial working memory task in healthy young participants, compared to amber light exposure. Similarly, Killgore et al. (2020) [14] observed that exposure to blue light resulted in a higher percentage of correct response and neural efficiency during a cognitive interference task than amber light. However, research on the effects of blue light on auditory working memory, particularly those involving the phonological loop, remains limited. Given the distinct neural substrates and processing characteristics of phonological loop compared to those of visuospatial systems, auditory working memory is anticipated to exhibit different sensitivities to blue light exposure. Understanding these effects is expected to provide valuable insights for optimizing learning environments and developing targeted cognitive interventions, especially in language- and communication-related domains.

Notably, Okamoto and Nakagawa (2016) [20] investigated the effects of blue versus green light on cortical oscillatory activity, as a neural process associated with working memory [21], during an auditory working memory task. They found that blue light exposure significantly increased event-related synchronization responses during the memory encoding phase compared to green light. Importantly, this effect was observed 20–30 min after the onset of blue light exposure, suggesting a delayed cognitive enhancement following blue light stimulation. These findings raise important questions regarding the temporal dynamics of blue light effects on cognition—specifically, whether the observed benefits emerge primarily after exposure or also during exposure. Although several studies have focused on post-exposure effects [10, 14, 22, 23], the cognitive impact of blue light during exposure remains poorly understood.

Taken together, this study aimed to investigate blue light effects on auditory working memory tasks involving the phonological loop, both during- and post-exposure. We hypothesized that blue light would enhance auditory working memory performance in both phases.

## Methods

### Participants

Sixteen healthy university students (14 males and 2 females, mean age: 21.38 ± 1.08 years) were recruited for this study. All participants were right-handed native speakers of Japanese with no history of psychiatric or sleep disorders, cognitive impairments, sensory deficits (vision or hearing), or use of medications affecting the central nervous system or sleep patterns. Prior to the study, participants maintained regular sleep schedules, with bedtimes between 10:00 p.m. and 1:00 a.m. and wake-up times between 6:00 a.m. and 9:00 a.m. Participants were not informed of the specific hypotheses of the study. This study was approved by the Ethics Committee of Hokkaido Bunkyo University in Japan. Informed consent was obtained from all participants, who were also compensated for their participation in this study.

### Experimental procedures

**Fig. 1:**
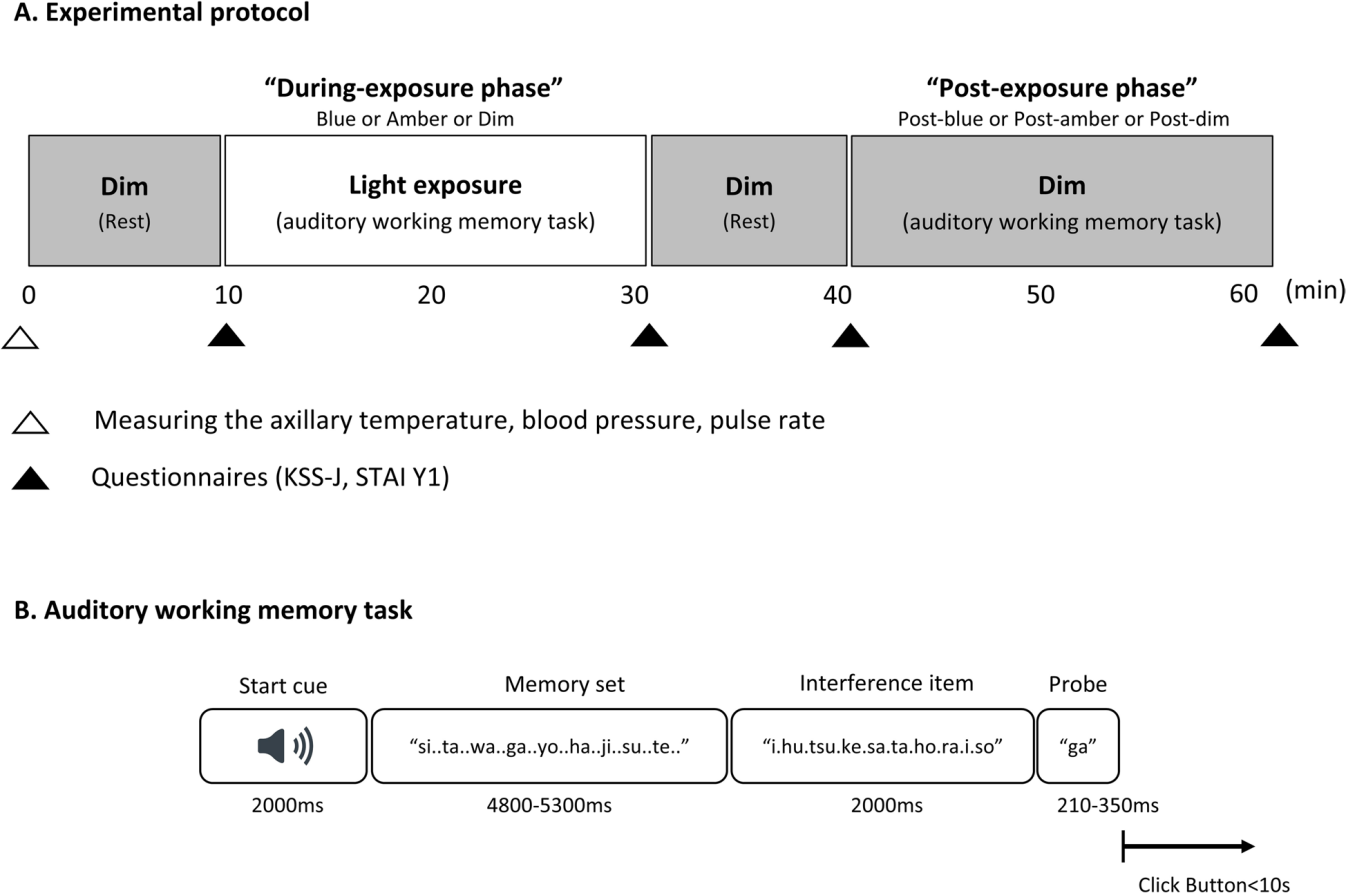
Experimental Protocol and Auditory Working Memory Task. **A**, Experimental protocol. Participants rested under dim light for 10 min before the task. They performed the auditory working memory task during exposure to one of three light conditions and again after a 10-min dim light rest. Sleepiness (KSS-J) and anxiety (STAI Y1) were assessed before and after each phase. **B**, Auditory working memory task. Participants memorized nine letters, followed by interference with meaningless Japanese monosyllables, then identified if a probe was part of the memory set. Trials began immediately after responses

Participants took part in three sessions, scheduled one week apart in a randomized crossover design. Each participant completed all sessions at the same time of day (10:00 a.m. or 2:00 p.m.), according to their preference, to minimize circadian effects on cognitive performance. The experimental paradigm was identical across sessions except for the three light exposure conditions (blue, amber, or dim, Fig. 1A), and the sequence of these conditions was randomized across participants to mitigate order effects. Participants were instructed to maintain their usual daily routine, including sleep schedules, prior to each session. They were also asked to report any significant deviations, and sessions were rescheduled if necessary. Upon arrival, participants provided written informed consent and underwent initial physiological assessments (axillary temperature, blood pressure and pulse). Following a 10-min rest in a dim light, participants completed an auditory working memory task for approximately 20 min under one of three light conditions, blue, amber, or dim light (referred to as the “During-exposure phase”). The duration of light exposure was set to 20 min based on findings by Okamoto and Nakagawa (2016) [20], who reported significant changes in cortical oscillatory activity beginning around 20 min after the onset of blue light exposure. Following another 10-min rest under dim light, they repeated a similar auditory working memory task for approximately 20 min under dim light (referred to as the “Post-exposure phase”). Each phase consisted of 100 trials, with a 1-min break after the first 50 trials. Subjective sleepiness was assessed before and after each phase using the Japanese version of the Karolinska Sleepiness Scale (KSS-J) [24, 25], and state anxiety was evaluated using the Japanese version of the State-Trait Anxiety Inventory Form Y1 (STAI-Y1) [26, 27].

### Experimental conditions

**Fig. 2:**
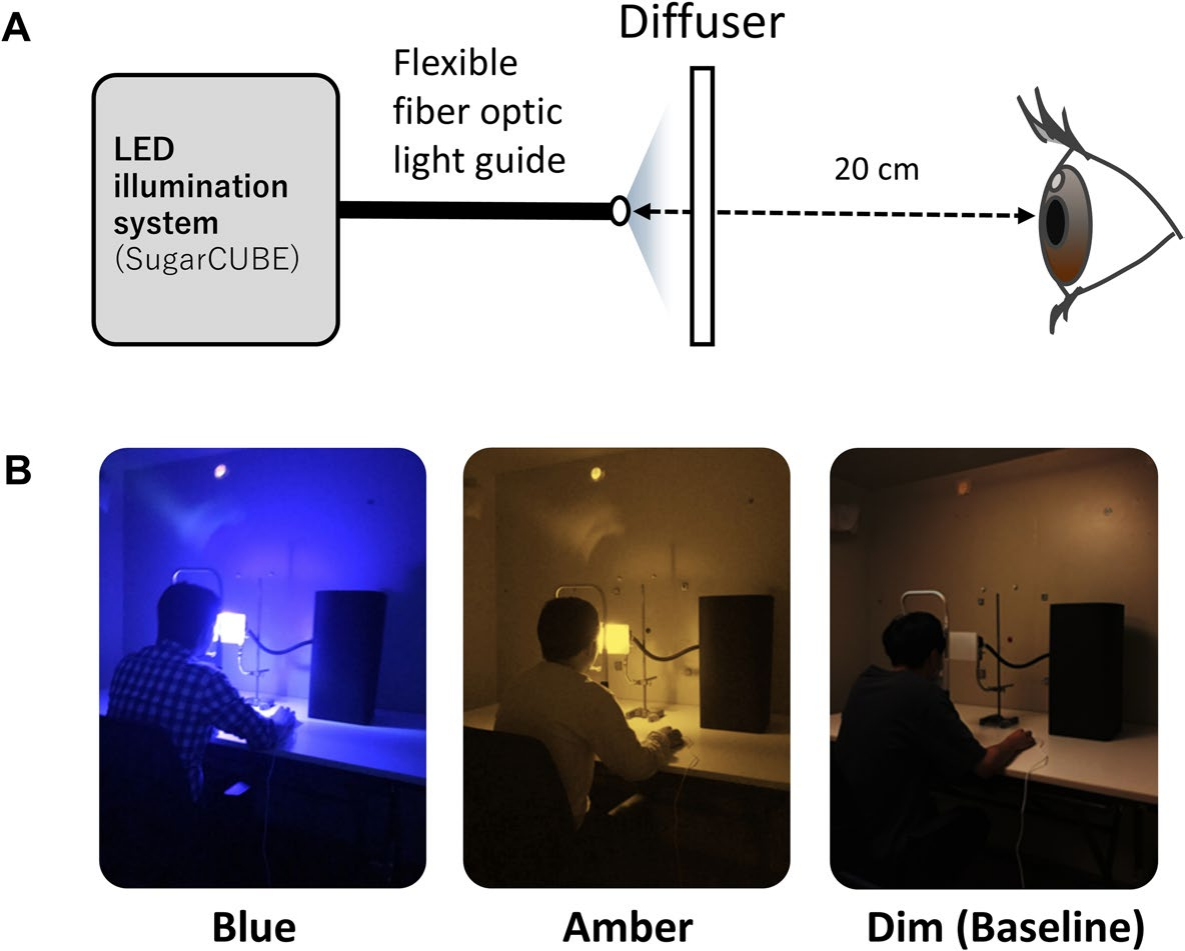
Experimental setup and images of light conditions. **A**, Schematic illustration of the LED-based lighting system (SugarCUBE) and participant's viewing position. **B**, Images of the three light conditions: blue light (left), amber light (middle), and dim light (right)

Figure 2 shows the experimental setup and images of light conditions. Light stimuli were delivered using an LED source (SugarCube, Edmund Optics, NJ) connected to a flexible fiber optic light guide (Edmund optics, NJ). Bandpass filters were placed at the tip of the light guide to produce blue (λ_max_ = 476 nm with a half-bandwidth of 10 nm) and amber (λ_max_ = 580 nm with a half-bandwidth of 10 nm) light. The photon densities of the blue and amber light at eye level were 13.8 and 13.5 log photons/s⁻^1^·cm⁻^2^, respectively [28]. Detailed radiometric and photometric properties are provided in Table 1. Amber light is known to have relatively limited activation of ipRGCs [17], and has therefore often been used as a control condition to distinguish the physiological and cognitive effects specific to blue light exposure [11, 23, 29]. The horizontal distance from the filter to the subject's eyes was maintained at 20 cm. To ensure consistent stimulus delivery, the horizontal distance from the filter to the participant’s eyes was fixed at 20 cm. A diffuser was placed 4 cm in front of the filter to ensure even light distribution across the visual field. Head position was stabilized using a chin rest (RP-HVY-CR, Zero C Seven, Japan).

**Table 1:** Radiometric and photometric quantities

	**Blue light**	**Amber light**	**Dim light**
peak wavelength of light [nm]	476	580	-
log photon irradiance [/s-1.cm-2]	13.8	13.5	-
illuminance [lx]	21.84	61.65	< 5
melanopic EDI [lx]	169.68	2.87	-

*EDI* equivalent daylight (D65) illuminance

### Measurements

The auditory working memory task performance was assessed using a modified version of the Sternberg working memory task [30], a validated tool for assessing working memory capabilities. During the test, participants responded to whether a probe item was present in their auditory memory set by clicking a button (Fig. 1B). The auditory stimulus consisted of a Japanese character voiced by a female and played through computer speakers. The letters were selected from 20 non-meaningful monosyllables used in the speech discrimination test defined by the Speech Discrimination Test Committee of the Japanese Society of Audiology [31]. Each memory set consisted of 9 letters, randomly selected from the 20 letters, with the restriction that each letter appeared only once in the same memory set. To minimize learning effects, a different letter combination was used in each trial. Additionally, interference items included 10 meaningless monosyllables, different from the characters used in the memory set. A probe item was sounded immediately following the interference item, and the subsequent trial commenced as soon as participants responded by pressing the button.

Changes in subjective anxiety state were assessed using the STAI Japanese version [27]. This questionnaire is a quantitative psychological assessment of the degree of anxiety using two scales (Y1 scale for state anxiety; Y2 scale for trait anxiety) [26]. In this study, only state anxiety was assessed to focus on the temporary anxiety state immediately before and after each experimental phase. The aggregate scores range from 20 to 80. A score below 41 on the State Anxiety Scale has been reported as a moderate level of healthy adults in Japan [32]. Additionally, the KSS-J was used to assess subjective sleepiness [25]. The KSS is a widely used 9-point scale that measures momentary (state) sleepiness, with scores ranging from 1 (“extremely alert”) to 9 (“very sleepy, fighting sleep”). The Japanese version has demonstrated sufficient validity and is regarded as a reliable tool for evaluating transient sleepiness in experimental contexts [25]. Participants completed the KSS-J immediately before and after each phase to assess changes in subjective alertness.

### Statistical analysis

We conducted statistical analysis using R version 3.5.3 (R Core Team, 2019). A one-factor repeated measures ANOVA was performed on the accuracy and response time of the working memory task for each phase, with the light condition as the independent variable. The Bonferroni test was used for post-hoc analysis. For subjective sleepiness and anxiety states in any of the phases, we calculated the changes in scores from before to after the phase and conducted tests with light condition as the independent factor. Following the assessment of normality using the Shapiro–Wilk test, a one-way repeated measures ANOVA was conducted on the subjective anxiety state, followed by Bonferroni-corrected paired t-tests and Tukey's HSD tests. For subjective sleepiness, the Friedman test was used due to the lack of confirmed normality in the data. The significance level was set at *p* < 0.05.

## Results

### Descriptive and confounding variables

One participant was excluded from the analysis for falling asleep during the experiment. Data from 15 participants (13 males and 2 females, mean age: 21.47 ± 1.06 years) were included in the statistical analysis. According to their self-reported sleep diaries, the average sleep durations did not differ significantly between the three light conditions (Table 2). Physical status data, including axillary temperature, blood pressure, and pulse, did not show significant differences among the three light conditions (Table 2).

**Table 2:** Physiological and sleep parameters (*n* = 15) Mean ± SE

	Blue light	Amber light	Dim light	*p* value
Axillary temperature [℃]	36.54 (0.09)	36.59 (0.06)	36.57 (0.08)	n.s
SBP [mmHg]	108.87 (3.41)	107.20 (3.33)	104.93 (3.98)	n.s
DBP [mmHg]	77.40 (2.32)	78.60 (2.88)	73.61 (2.41)	n.s
Pulse rate [beat per minute]	82.73 (3.56)	80.60 (3.20)	79.87 (3.19)	n.s
Sleep duration the night before [hours]	7.27 (0.45)	7.25 (0.42)	8.30 (0.33)	n.s

*SE* Standard Error, *SBP* Systolic Blood Pressure, *DBP* Diastolic Blood Pressure, *n.s.* not significant

### Working memory performance task

**Fig. 3:**
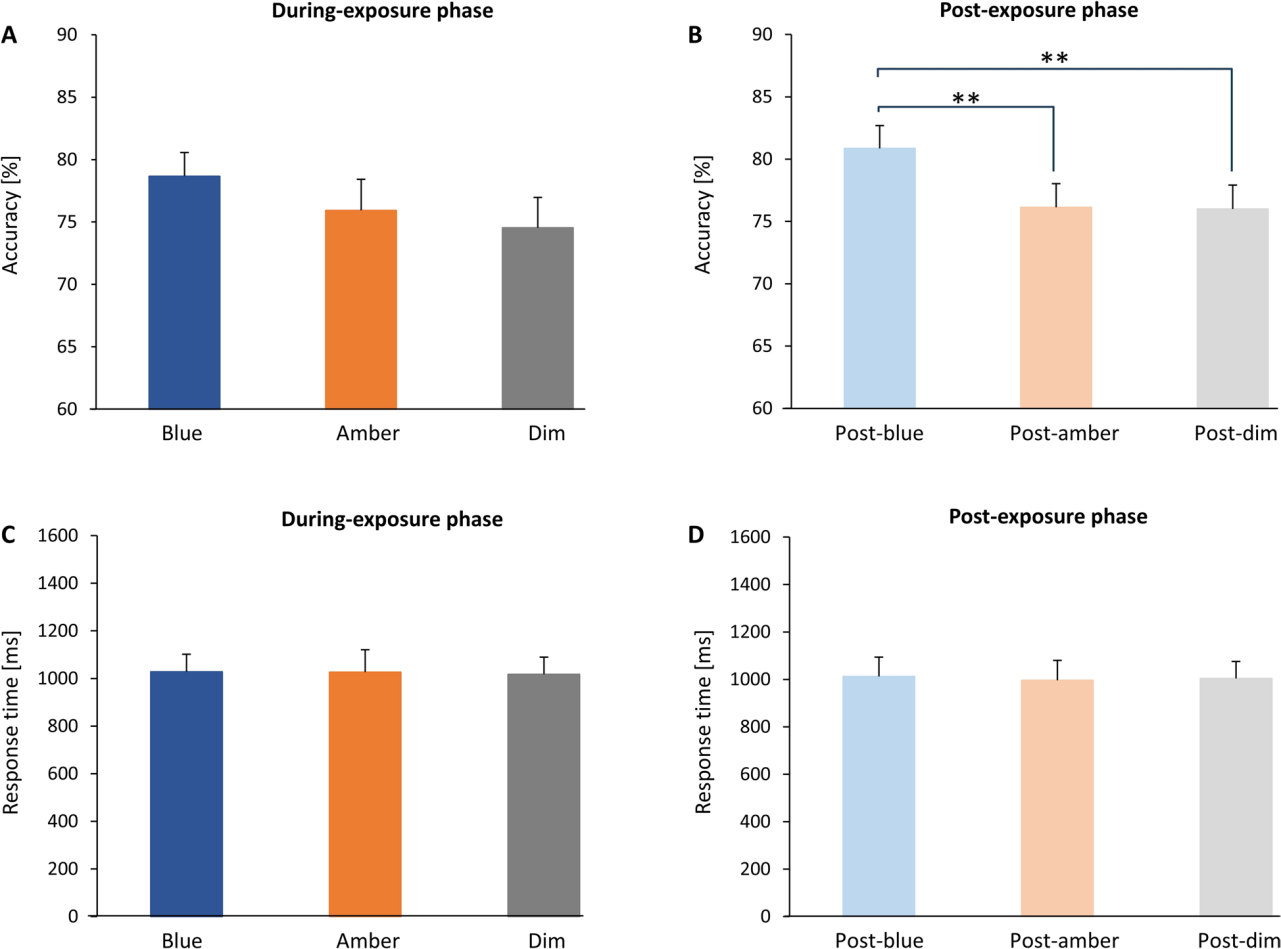
Accuracy and Response Time in Auditory Working Memory Task. **A**, No significant effect of light condition on accuracy during the During-exposure phase. **B**, Accuracy was significantly higher under blue light compared to amber and dim light during the Post-exposure phase. **C** & **D**, No significant effect of light condition on response time in either phase. Error bars represent + 1 SE. *p < 0.05; **p < 0.01

A one-way repeated measures ANOVA of the accuracy for the working memory task in the During-exposure phase showed no main effect of light condition (F(2, 28) = 2.70, *p* = 0.085, η*p*^2^ = 0.162; Fig. 3A). In the Post-exposure phase, there was a significant main effect of light condition on working memory task accuracy (F(2, 28) = 12.25, *p* < 0.001, η*p*^2^ = 0. 467; Fig. 3B). The accuracy for the working memory task was significantly higher in the blue light exposure condition compared to both the amber light exposure and dim light exposure conditions (blue light exposure vs. amber light exposure, *p* < 0.01, Cohen's d = 0.66, 95% CI [−0.11, 1.43]; blue light exposure vs. dim light exposure, *p* < 0.01, Cohen's d = 0.67, 95% CI [−0.10, 1.44]; amber light exposure vs. dim light exposure, *p* = 1.00, Cohen's d = 0.01, 95% CI [−0.73, 0.77]; Fig. 3B). No main effect of light condition on response time for the working memory task was found in each phase (During-exposure phase: F(2, 28) = 0.09, *p* = 0.92, η*p*^2^ = 0.004; Post-exposure phase: F(2, 28) = 0.06, *p* = 0.95, ηp^2^ = 0.009; Figs. 3C, D).

### Subjective anxiety state

**Fig. 4:**
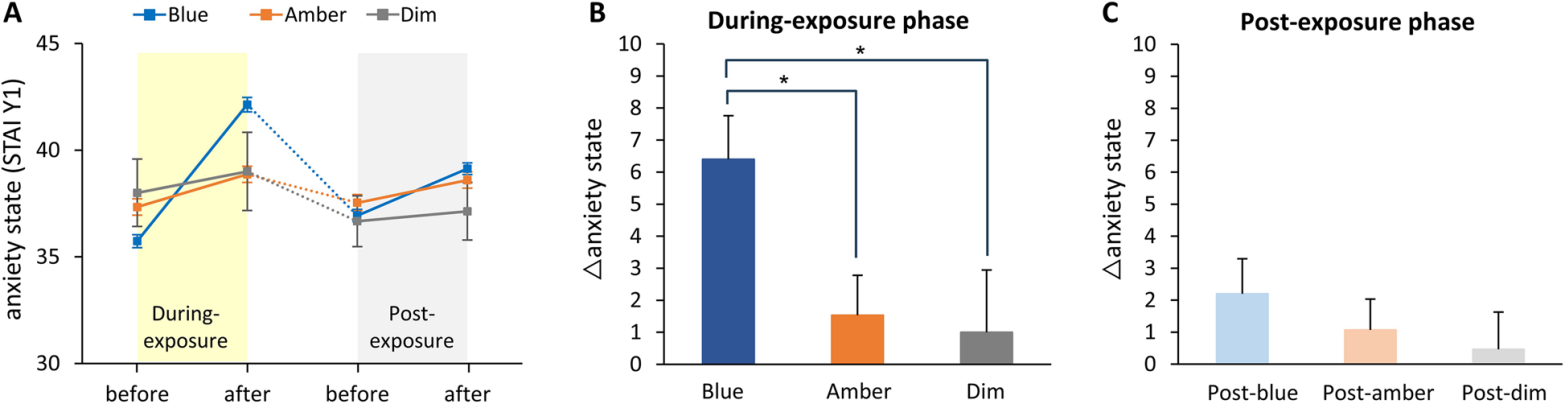
Subjective Anxiety State Scores. **A**, Scores before and after each phase. **B**, Changes during the During-exposure phase. Blue light increased anxiety levels significantly more than amber and dim light, with no difference between amber and dim light. **C**, Changes during the Post-exposure phase. No significant effect of light conditions on anxiety changes. Error bars represent + 1 SE. *p < 0.05; **p < 0.01

The subjective anxiety state scores before and after each phase are shown in Fig. 4A. In the During-exposure phase, the analysis revealed a significant main effect of light condition on changes in subjective anxiety state (F(2, 28) = 6.04, *p* < 0.01; Fig. 4B). Subsequent pairwise comparisons showed that participants exhibited significantly higher state anxiety levels under blue light compared to amber light (paired t-test, Bonferroni-corrected; *p* = 0.013, Cohen's d = 0.96, 95% CI [0.17, 1.75]; Fig. 4B) and compared to dim light (*p* = 0.027, Cohen's d = 0.83, 95% CI [0.05, 1.61]; Fig. 4B). However, there was no significant difference in changes of subjective anxiety state levels between amber and dim light conditions (*p* = 1.000, Cohen’s d = 0.08, 95% CI [−0.66, 0.83]; Fig. 4B). In the Post-exposure phase, there was no main effect of light condition on changes in subjective anxiety state (F(2, 28) = 0.615, *p* = 0.55; Fig. 4C).

### Subjective sleepiness

**Fig. 5:**
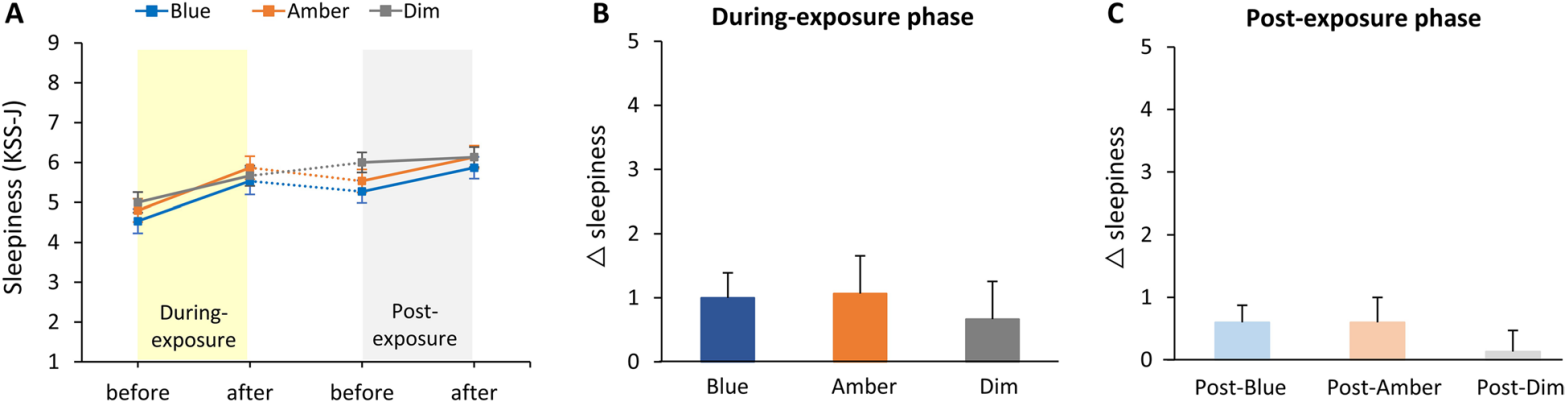
Subjective Sleepiness Scores. **A**, Scores before and after each phase. **B** & **C**, No significant effect of light condition on changes in sleepiness during either phase. Error bars represent + 1 SE. *p < 0.05; **p < 0.01

The subjective sleepiness levels before and after each phase are shown in Fig. 5A. No significant main effect of light condition on changes in subjective sleepiness levels was found in either phase (During-exposure phase: Friedman chi-squared = 0.82, df = 2, *p* = 0.66; Fig. 5B; Post-exposure phase: Friedman chi-squared = 1.38, df = 2, *p* = 0.50; Fig. 5C).

## Discussion

This study investigated the during- and post-exposure effects of blue light on auditory working memory tasks involving the phonological loop. Accuracy was significantly higher in the post-exposure phase under blue light compared to amber and dim light. These results suggest that blue light enhances auditory working memory performance, with effects that emerge over time. These results are consistent with previous findings associating blue light exposure with increased alertness and cognitive performance [9–11, 14, 22, 23]. However, no significant differences were observed in response time or subjective sleepiness across light conditions. Although session timing varied across participants, each participant completed all sessions at the same time of day to control for intra-individual circadian variation. This within-subject consistency likely minimized the influence of sleepiness on task performance and supported the interpretation that blue light may selectively enhance cognitive accuracy rather than processing speed.

The observed post-exposure improvement reflects sustained activation of ipRGCs, which remain responsive even after light exposure [18, 33, 34]. This is consistent with findings by Okamoto and Nakagawa (2016) [20], who reported enhanced cortical activity related to attention and memory 20–30 min following the onset of blue light exposure. The mechanism underlying the effects of blue light exposure on cognitive function has been shown to involve its influence on neural pathways in the PFC through various interconnected processes [10, 14, 23, 35]. Recent studies suggest that blue light enhances functional connectivity between the left dorsolateral PFC and regions across the temporal, parietal, and occipital lobes, which is thought to contribute to improved performance on tasks related to spatial attention, motion perception, and working memory [35]. Additionally, fMRI studies have shown that even brief exposure to blue light increases activity in a wide range of brain regions, including the hippocampus, thalamus, amygdala, and brainstem, with notable activation of the locus coeruleus (LC), a key structure involving in regulating arousal and attention [36]. Blue light has been shown to activate the LC via norepinephrine signaling, enhancing task-related attention while suppressing irrelevant neural activity [14]. Animal studies have further demonstrated that the LC-norepinephrine system dynamically modulates brain states, with tonic firing activating higher-order cognitive regions and burst firing engaging sensory regions [37]. By stimulating the LC, blue light appears to shift network activity in accordance with situational demands, thereby influencing cognitive performance.

In the during-exposure phase, however, no significant effects of blue light were observed, suggesting that the acute cognitive impacts of blue light might be limited. Few studies have directly assessed cognitive functioning during light exposure [20], and the absence of acute effects in the present study may be attributed to factors such as the relatively low irradiance, short exposure duration, or task-specific cognitive demands. While mechanisms such as melatonin suppression and increased physiological arousal are well-documented, their influence on real-time cognitive processing during light exposure remains unclear [10, 22, 29]. It is therefore plausible that a minimum threshold of exposure is needed for blue light to exert measurable effects, which emerge following a delay.

The STAI Y1 scores in this study remained within the normative range for healthy Japanese adults [27, 33]. However, a transient increase in anxiety levels was observed during blue light exposure, potentially reflecting activation of the sympathetic nervous system. Previous studies have shown that blue light can elevate physiological arousal, including increased heart rate and blood pressure [29], and that activation of ipRGCs has been reported to induce heightened vigilance and anxiety-like behavior in animal models [38]. Recent neuroimaging studies have shown that blue light enhances activity in brain regions involved in emotional regulation, such as the amygdala and PFC, potentially explaining its transient anxiogenic effects [24]. Further research is needed to clarify the mechanisms linking blue light and anxiety.

Given the interaction between blue light, stress, and cognition, the transient increase in anxiety may have influenced task performance. Acute stress is known to either enhance or impair cognition depending on individual brain network profiles, such as dominance of the executive control network or salience network [39, 40]. Blue light is suggested to induce an acute stress response, potentially increasing anxiety while simultaneously enhancing cognitive performance via activation of higher-order circuits [36, 37, 39, 40]. This interaction has the potential to modulate cognitive performance, depending on individual differences in brain network profiles and stress reactivity, thereby highlighting the need for personalized approaches in future research.

Several limitations should be acknowledged. First, although participants were limited to healthy university students with no reported history of neurological or psychological disorders, no standardized baseline cognitive assessment was conducted prior to participation. Second, physiological and neurophysiological measures should be incorporated in future studies to objectively support behavioral outcomes, including task performance and self-reported anxiety. Third, objective monitoring of sleep–wake states is warranted, given that one participant was observed to fall asleep during a session. Fourth, while a 10-min break was implemented before post-exposure assessment, a longer interval is essential to more accurately capture the delayed effects of blue light exposure. Fifth, the relatively low intensity of blue light used in this study necessitates further investigation into intensity-dependent responses. Seventh, the small sample size and gender imbalance limited the generalizability of findings and precluded analysis of sex differences. Future studies should aim to recruit larger, gender-balanced samples, especially given previous evidence demonstrating that sex differences in light sensitivity can influence brightness perception, vigilant attention, and sleep physiology [41]. Moreover, the cognitive effects of blue light observed in this study may be limited to younger adults. Age-related changes such as reduced melatonin regulation, yellowing of the ocular lens, and alterations in circadian rhythm [42] may diminish sensitivity to blue light, potentially leading to different outcomes in older populations.

Finally, while blue light has shown to confer cognitive benefits in healthy adults, its effects on children, particularly those with neurodevelopmental disorders, remain unclear. Children with attention-deficit/hyperactivity disorder (ADHD) often struggle with working memory due to deficits in attention regulation and cognitive control. Although interventions targeting working memory have demonstrated effectiveness in this population [43], the potential use of blue light as a non-invasive cognitive enhancement tool for both typically developing and neurodivergent children requires further investigation.

## Conclusions

This study demonstrated that blue light exposure enhanced auditory working memory accuracy in the post-exposure phase, suggesting a delayed cognitive effect. This benefit appeared to be independent of subjective sleepiness and response time, indicating that blue light may enhance accuracy without affecting vigilance or processing speed. Possible underlying mechanisms include activation of ipRGCs, modulation of cortical networks, and transient emotional arousal, which may act through both neural and affective pathways. Although the findings are promising, they should be interpreted with caution due to methodological limitations, including a small sample size, low stimulus intensity, and limited demographic diversity. Future studies should refine exposure parameters, consider individual differences in age and brain network profiles, and explore broader applications in clinical and educational contexts. These preliminary results provide valuable insights for future strategies aimed at optimizing lighting environments and developing cognitive interventions. However, their practical significance needs to be established through validation in larger and more diverse populations.

## Data Availability

No datasets were generated or analysed during the current study.

## Abbreviations

ipRGCs: Intrinsically photosensitive retinal ganglion cells
PFC: Prefrontal cortex
KSS-J: Japanese version of the Karolinska sleepiness scale
STAI Y1: State-trait anxiety inventory form Y1
ANOVA: Analysis of variance
HSD: Honestly significant difference
CI: Confidence interval
EDI: Equivalent daylight (D65) illuminance
SBP: Systolic blood pressure
DBP: Diastolic blood pressure
LC: Locus coeruleus
ADHD: Attention-deficit/hyperactivity disorder
